# A Bacteriophage DNA Mimic Protein Employs a Non-specific Strategy to Inhibit the Bacterial RNA Polymerase

**DOI:** 10.3389/fmicb.2021.692512

**Published:** 2021-06-02

**Authors:** Zhihao Wang, Hongliang Wang, Nancy Mulvenna, Maximo Sanz-Hernandez, Peipei Zhang, Yanqing Li, Jia Ma, Yawen Wang, Steve Matthews, Sivaramesh Wigneshweraraj, Bing Liu

**Affiliations:** ^1^BioBank, The First Affiliated Hospital of Xi’an Jiaotong University, Xi’an, China; ^2^MRC Centre for Molecular Bacteriology and Infection, Imperial College London, London, United Kingdom; ^3^Department of Pathogen Biology and Immunology, School of Basic Medical Sciences, Xi’an Jiaotong University, Xi’an, China; ^4^Instrument Analysis Centre of Xi’an Jiaotong University, Xi’an, China

**Keywords:** bacteriophage, RNA polymerase, DNA binding ability, intrinsic disordered protein, DNA mimic protein

## Abstract

DNA mimicry by proteins is a strategy that employed by some proteins to occupy the binding sites of the DNA-binding proteins and deny further access to these sites by DNA. Such proteins have been found in bacteriophage, eukaryotic virus, prokaryotic, and eukaryotic cells to imitate non-coding functions of DNA. Here, we report another phage protein Gp44 from bacteriophage SPO1 of *Bacillus subtilis*, employing mimicry as part of unusual strategy to inhibit host RNA polymerase. Consisting of three simple domains, Gp44 contains a DNA binding motif, a flexible DNA mimic domain and a random-coiled domain. Gp44 is able to anchor to host genome and interact bacterial RNA polymerase *via* the β and β′ subunit, resulting in bacterial growth inhibition. Our findings represent a non-specific strategy that SPO1 phage uses to target different bacterial transcription machinery regardless of the structural variations of RNA polymerases. This feature may have potential applications like generation of genetic engineered phages with Gp44 gene incorporated used in phage therapy to target a range of bacterial hosts.

## Introduction

Bacteriophages, or bacterial viruses, have evolved distinct mechanisms to take over various host biological processes for effective reproduction. Phage enters the host *via* specific receptors on the surface of bacteria, which limits its host range (Koskella and Meaden, 2013). After entering the host, phage produces many proteins that interact with bacterial key enzymes to inhibit or modify related biological activities (Salmond and Fineran, 2015). RNA polymerase is the predominant target for the phage to utilize the bacterial transcription machinery in the early stage of invasion and to inhibit the host RNA polymerase activity in the later stages (Krupp, 1988). The majority of the bacterial transcription studies are carried out on *Escherichia coli*, and studies on prototypical lytic phage of T7 and T4 of Gram-negative model bacterium—*E. coli* and their RNA polymerase (RNAP) inhibitory proteins, Gp0.7, Gp2, and Gp5.7 (T7) and AsiA (T4) shed lights on how phages modulate host RNAP activity (Lambert et al., 2004; Severinova and Severinov, 2006; Bae et al., 2013; Tabib-Salazar et al., 2017, 2018, 2019). Understanding the molecular mechanisms of phage antibacterial proteins and their interactions with RNAP has inspired research into new antibacterial compounds or treatments for Gram-negative bacteria (Sunderland et al., 2017).

Different from the core *E. coli* RNAP complex which consists of two α subunits and single β, β′, and ω subunits (α_2_ββ′ω), *Bacillus subtilis* has a α_2_ββ′δω_1_ω_2_ subunit composition (Wiedermannova et al., 2014). As a result, the study of Gram-positive bacterial phages has been lagging behind its counterpart for a long time and in need of revamp to stimulate the search for new antibacterial approaches (Pires et al., 2016). SPO1 is a lytic phage that infects *B. subtilis* (Stewart, 2018). A cluster of genes in the SPO1 genome, called the host takeover module, which are expressed early during the infection process, encode proteins associated with modulation and/or inhibition of bacterial processes for efficient takeover of the bacterial cell for the production of viral progeny (Stewart et al., 1998; Mulvenna et al., 2019; Zhang et al., 2019; Wang et al., 2020). The expression of SPO1 genes are temporally coordinated and products of host takeover genes *gp44*, *gp50*, and *gp51* have been previously reported to be essential for the transition from early to middle gene expression and complete shutoff of host DNA, RNA, and protein synthesis (Sampath and Stewart, 2004). The expression of recombinant Gp44, a 27 kDa negatively charged protein, in either *B. subtilis* or *E. coli*, results in inhibition of host DNA, RNA and protein synthesis and eventual demise of the bacterial cell (Wei and Stewart, 1993). As RNA synthesis was found to be most affected by expression of Gp44, Gp44 was considered to be an RNAP interacting protein. Moreover, the central region of Gp44 shows remarkable sequence resemblance to the RNAP binding region of σ^54^—the nitrogen-limitation sigma factor (Tintut et al., 1994). Gp44 was reported to act as a competitor of the DNA interaction with the β subunit (Wei and Stewart, 1995). Gp44 is an also very acidic protein, therefore it is conceivable that it can adopt biophysical features of DNA to compete.

DNA mimicry by proteins is a strategy often used by viruses, prokaryotic and eukaryotic cells to inhibit or interfere with the activity of DNA interacting proteins (Bochkareva et al., 2005; Hegde et al., 2005; Wang et al., 2008). DNA mimic proteins (DMPs) often function by directly occupy the DNA binding cavity of the cognate substrate proteins to inhibit their DNA binding activity (Putnam and Tainer, 2005; Dryden, 2006; Dryden and Tock, 2006). For effective DNA mimicry, DMPs must display a conformation resembling DNA and have a localized negative charge distribution. While DNA also carries genetic information in all cellular forms of life and some viruses, DMP does not contain any genetic information. Furthermore, although DNA double helix structure has a degree of flexibility (Levitt, 1982), DMPs reported so far do not have DNA-like structural flexibility but rather rigid secondary or tertiary protein structure features (Tucker et al., 2014).

Here, we have used multidimensional nuclear magnetic resonance (NMR) spectroscopy and molecular dynamics to elucidate structural features of Gp44. It contains a DNA binding motif, a DNA mimic domain and a random-coiled domain—a combination never found in any known mimic proteins to our knowledge (Wang et al., 2014). And we proposed a new model in which Gp44 interacts with β and β′ subunit of RNAP to interfere with bacterial RNAP activity during SPO1 development.

## Materials and Methods

### Protein Expression and Purification

SPO1 gene 44 and the truncated constructs were PCR amplified from SPO1 genomic DNA using pET EK/LIC primers for ligation independent cloning and cloned into pET-46 EK/LIC for adding N terminal His-tag and pDE2 vector for C-terminal His-tag or tag free constructs. Three Gp44 constructs: (1–55), (1–122), and full length (1–237) were made by nickel affinity purification from *E. coli* strain BL21(DE3). The culture of BL21(DE3) cells contains pET-46 Gp44 construct, was grown at 37°C to OD_600_ of ∼0.6 and induced by 1 μM of IPTG. The cells were left to continue growing at 37°C for 4 h before harvesting. The cell pellet was re-suspended in binding buffer (50 mM NaH_2_PO_4_, pH 8.0, 0.3 M NaCl, and 5 mM DTT) containing cocktail of protease inhibitors and lysed by sonication. The cleared cell lysate was loaded onto a His-Trap HP column (GE Healthcare Life Sciences), which was connected to AKTA pure chromatograph machine. The purified protein was eluted over a 50 ml gradient of 0–100% Elution buffer (Binding buffer + 1 M imidazole pH 8.0) according to manufacturer’s instructions. The purified protein was dialyzed into storage buffer (50 mM NaH_2_PO_4,_ pH 6.8, 250 mM NaCl, 1 mM DTT) and concentrated for NMR studies. Expression and purification of β and β′ subunits were using pDE1 vector and followed the same protocol described above. Expression and purification of recombinant *B. subtilis* RNA polymerase followed the protocol described by Yang and Lewis (2008).

### Pull-Down Assays

For His-tag protein pull down assay, the Ni-NTA column was first equilibrated with five column volumes of lysis buffer (10 mM imidazole) then the His-tagged protein was applied into the column. The column was subsequently washed with five column volumes of lysis buffer before applying supernatant of *B. subtilis* (strain 168) cell lysate which was harvested at 0.8 of OD_600_. The column was washed with 10 column volumes of lysis buffer before samples were eluted with 50 μl of Laemmli 2x concentrate SDS Sample Buffer. Samples were then boiled for 5 min prior to analysis by SDS-PAGE.

### NMR Structure Determination

Nuclear magnetic resonance spectra were collected at 310K on Bruker DRX600 and DRX800 spectrometers equipped with cryo-probes. Spectral assignments were completed using our in-house, semi-automated assignment algorithms and standard triple-resonance assignment methodology. H_α_ and H_β_ assignments were obtained using HBHA (CBCACO)NH and the full side-chain assignments were extended using 3D HCCH-TOCSY and (H)CCH-TOCSY experiment. Three-dimensional ^1^H-^15^N/^13^C NOESY-HSQC (mixing time 100 ms at 800 MHz) experiments provided the distance restraints used in the final structure calculation. The ARIA protocol was used for completion of the NOE assignment and structure calculation. The frequency window tolerance for assigning NOEs was ±0.025 ppm and ±0.03 ppm for direct and indirect proton dimensions and ±0.6 ppm for both nitrogen and carbon dimensions. The ARIA parameters p, Tv, and Nv were set to default values. 110 dihedral angle restraints derived from TALOS were also implemented. The 10 lowest energy structures had no NOE violations greater than 0.5 Å and dihedral angle violations greater than 5°. The structural statistics are shown in Supplementary Table 1.

### NMR Titration

One equivalent of β or β′ subunit of either *E. coli* or *B. subtilis* was added the unlabeled full length Gp44 to perform the 1D titration. Maximal twofolds of protein were added to Gp44 in order to reach the end of the reaction. For the DNA titration, double stranded DNA fragments was added to ^15^N labeled Gp44 constructs according to stoichiometric ratio to perform NMR titration. Maximal fivefold dsDNA was added to Gp44 in order to reach the end of the reaction.

### ITC

Isothermal titration calorimetry (ITC) experiments were performed on a MicroCal PEAQ-ITC Instrument (Microcal) at 25°C using the dialysis buffer described above. β or β′ subunit 30 μM in the cell was titrated with 300 μM Gp44 in the syringe *via* 19 injections with 2 μL each at 120 s interval. The raw data were integrated, normalized for the molar concentration and analyzed using MicroCal PEAQ-ITC Analysis Software.

### Bacterial Growth Attenuation Assays

Start cultures were grown at 37°C, shaking at 700 rpm. for 6–7 h in a plate incubator by directly inoculating a colony from a freshly transformed Luria agar plate into 200 μl of Luria broth (LB) medium containing 100 μg carbenicillin ml^–1^ into a 96-well microtiter plate (Corning). The start cultures were diluted 1:100 in a final volume of 200 μl of fresh LB medium containing 100 μg carbenicillin ml^–1^ and incubated at 30°C, shaking at 500 rpm. The expression of Gp44 constructs was induced at OD_600_ of ∼0.2–0.25 by adding 0.1 μm of isopropeyl-β-D-thiogalactoside (IPTG) for *E. coli* strains DH5α and ATCC35218. The experimental growth curves were also performed in 96-well microtiter plates in a Cytation3 multi-well plate reader (BioTek). At least three biological and technical replicates were performed for each growth curve.

### Bacterial Two-Hybrid Assay

Bacterial two-hybrid assay was performed as previously described (Rao et al., 2009) with minor modifications. Briefly, GP44 (or mutant) and RNAP subunit were first subcloned into pAC and pRB constructs, respectively. KS1 reporter strains cells were then co-transformed with the indicated pAC and pBR derived plasmids and grown in LB containing 100 μg/ml carbenicillin, 35 μg/ml chloramphenicol and 50 μg/ml kanamycin. pAC and pRB empty vectors were used as negative control, while pAC-β flap and pRBL28 co-transformation were used as positive control. When the OD_600_ of the culture reached 0.3, cells were treated with indicated concentrations of isopropeyl-β-D-thiogalactoside (IPTG) for 1 h followed by 50 μM Fluorescein di-β-D-galactopyranoside (FDG) for another 1 h. β-galactosidase activity was assayed by measuring the fluorescence intensity of fluorescein with Cytation 3 plate reader equipped with 485 ± 20 nm excitation and 520 ± 25 nm emission filters.

### Molecular Dynamics Simulation

Two homologous conserved fragments were extracted from Gp44^56–122^ using RADAR (Li et al., 2015). The initial disordered 3D structures of the fragments were obtained from I-TASSER, and the starting structures were chosen after energy minimization. The fragments were solvated with water, in cubic box and Na^+^ and Cl^–^ ions were added to balance the charges. An improved CHARMM36 force field which was optimized to simulate both ordered and disordered proteins (Huang et al., 2017) were employed to model the protein molecules. MD simulations were performed using GROMACS 4.6.7 molecular dynamic engine (Hess et al., 2008). The fragments were constrained, and their energy minimization were carried out, followed by the 1 ns equilibration at NVT and NPT ensembles at 300K and 1 bar, respectively. The fragments were then simulated for 500 ns each, with protein snapshot saved every 500 ps (running parameters are summarized in Supplementary Table 3).

### Protein Identification by Mass Spectroscopy

Identification was done locally at instrument analysis center. Samples were digested with trypsin and desalted by a C18 ZipTip. Peptides were analyzed by Q-Exactive plus mass spectrometer (Thermo Fisher Scientific) coupled with an UltiMate 3000 RSLCnano system. Database search were performed by MaxQuant.

## Results

### Gp44 Interacts With β and β′ Subunits of Both *Escherichia coli* and *Bacillus subtilis* RNAP

**FIGURE 1:**
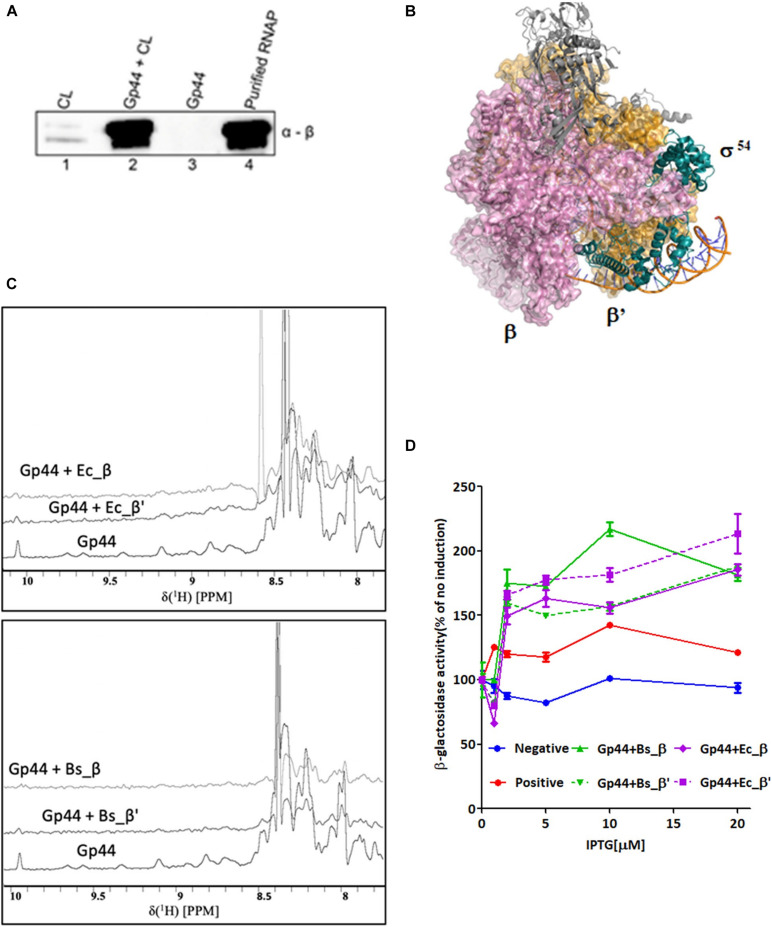
Gp44 interacts with β and β′ subunits of *B. subtilis* and *E. coli* RNAP. **(A)** Images of SDS-PAGE gel (left panel) and immunoblotting with anti RNAP β subunit antibody (right panel) showing results of the pull-down assay with His-tagged Gp44 and whole-cell extracts (WCL) of *B. subtilis.* **(B)** Complex structure of RNAP-σ^54^ holoenzyme initial transcribing complex in which σ^54^ interacts with RNAP *via* β and β′ subunits (PDB ID: 6GFW. σ^54^, β and β′ subunits are labeled respectively.) **(C)** ^1^H NMR spectra showing peak broadening effect when adding β and β′ subunits of *B. subtilis* and *E. coli* to Gp44. **(D)** BTH assay shows Gp44 interacts with β and β′ subunits of *E. coli* and *B. subtilis*.

To demonstrate Gp44 directly interacts with RNAP, we first expressed recombinant Gp44 fused with Histidine tag (His-tag) in *E. coli*. A pull-down assay using whole-cell extracts of exponentially growing *B. subtilis* cells expressing His-tagged Gp44 revealed a band around 120 kDa, which can be recognized by antibody against β subunit of *B. subtilis* RNAP and mass spectroscopy results confirmed that sample contains both β and β′ subunit (Figure 1A). To further localize the subunit(s) that Gp44 interacts with, we analyzed into the RNAP interacting region of σ^54^, which interacts with both β and β′ subunits (Figure 1B) and postulated that Gp44 might target the β and β′ subunits. We thus used 1D ^1^H NMR titration and a bacterial two-hybrid assay (BTH) to verify the hypothesis. In the NMR experiment, we successfully expressed and purified four recombinant subunits (β and β′ subunits of *E. coli* and *B. subtilis*) and titrated with Gp44 separately. After addition of any of the four subunits, all ^1^H spectra of Gp44 demonstrated peak broadening effects, suggesting a productive interaction (Figure 1C). Furthermore, we used ITC to determine the binding affinities (Supplementary Figure 1A). The K_*D*_s for the two interactions were determined at 3.46 ± 0.27 μM for Gp44-β and 4.08 ± 0.31 μM for Gp44-β′, respectively. These values were consistent with the peak broadening observed in the NMR titration experiments as both fell into the intermediate NMR timescale. Meanwhile, BTH assay results confirmed the NMR results, showing increases of β-glactosidase activity after IPTG induction of Gp44 and RNAP subunit expression (Figure 1D).

While RNAP of *E. coli* has five subunits—α_2_ββ′ω, that of *B. subtilis* has seven subunits α_2_ββ′δω_1_ω_2_ representing Gram-positive bacteria (Weiss and Shaw, 2015). As Gp44 is able to interact with both RNAP subunits regardless of structural variations suggests the possible existence of a new RNAP inhibitory mechanism.

### Domain Organization of Gp44

**FIGURE 2:**
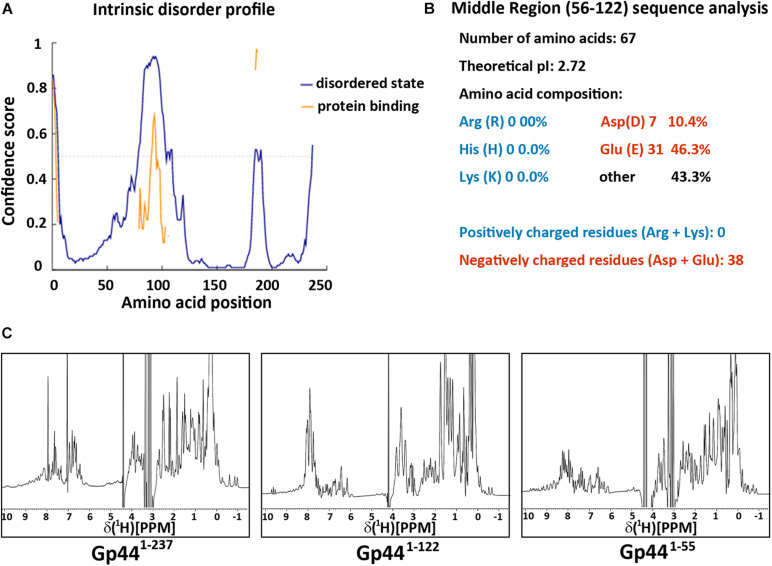
Gp44 has three distinctive domains. **(A)** Intrinsic disorder profile predicted by DISOPRED. Blue line stands for the likelihood of protein in the natural disordered state and yellow line indicates the possibility of protein binding **(B)** Primary structure analysis of the middle disorder domain (56–122), including its ultra-low pI and extreme negative charged amino composition. **(C)** 1D ^1^H spectra of three Gp44 constructs. From left to right: (Gp44^1– 237^), (Gp44^1– 122^), and (Gp44^1– 55^).

To understand the mechanism of Gp44 inhibition further, we employed various structural and biophysical tools for analysis. Despite its resemblance to σ^54^ in the central region (amino acids 56–122 of Gp44 to 44–113 of σ^54^) (Supplementary Figure 1B), the overall amino acid sequence of Gp44 only shares similarity among *Bacillus* phages in BLAST analyses. No homologous structures have been deposited in Protein Data Bank (PDB) and 3D structural prediction servers failed to produce meaningful predictions. As there are clusters of continuous negatively charged amino acids in its sequence, it is conceivable that Gp44 may not form regular secondary structure due to electrostatic repulsion in these regions. To assess the intrinsic disorder in this region, we use DISOPRED server (Ward et al., 2004) to identify regions in Gp44 that have increased propensity to be disordered. As shown in Figure 2A, the middle of Gp44 ranging from residue 56–122 (Gp44^56–122^) has a very high possibility of being disordered. Strikingly, this region consisted primarily of negatively charged amino acids where either aspartate or glutamate making up 57% of amino acids in this region (Figure 2B), which is more extreme than its counterpart in σ^54^ which carries 22% negatively charged amino acids. Notably, no positively charged amino acid residues (arginine, histidine, and lysine) were present in this region. Nevertheless, the percentage of charged amino acids in the rest of the sequence is also high in the rest of the sequence.

To identify the existence of any folded globular regions, we expressed and purified N-terminal histidine tagged (His-tagged) full length and truncated constructs of Gp44 and examined their foldedness by NMR. Dispersion in the ^1^H NMR spectrum of full-length Gp44 revealed the clear presence of a folded domain as well as unstructured region (Figure 2C). The spectrum of a C-terminally truncated construct (Gp44^1–122^) shows the folded domain is still present with only unstructured residues removed. After further removing central acidic region, the final construct (Gp44^1–55^) is fully folded and feasible for NMR structure determination.

Our full length recombinant Gp44, either tag free or carboxy-terminal (CT) His-tagged, fully inhibits the growth of both *E. coli* and *B. subtilis* as reported by Wei et al., suggesting the recombinant protein used in our NMR experiments is its natural functional state. As the presence of a functional protein ensures the comprehensive structural study of Gp44 based on the initial NMR structural analysis, we are able to divide it into three distinctive domains—a folded NT domain, a negatively charged middle domain and an unstructured CT domain.

### Amino Acid Residues 1–55 of Gp44 Interact With Double-Stranded DNA

**FIGURE 3:**
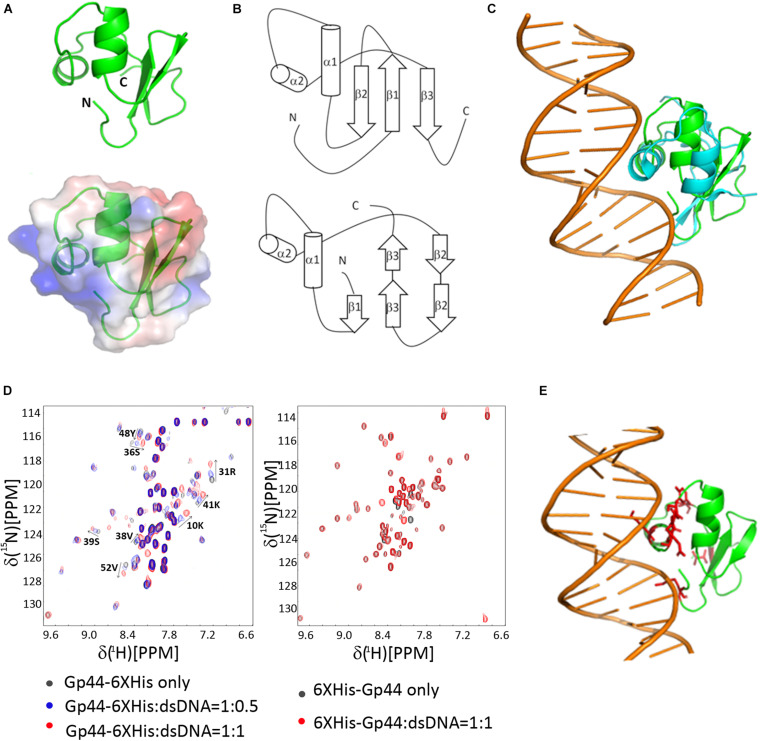
NMR-derived three-dimensional structure of Gp44NT and DNA interaction **(A)** A cartoon representation of Gp44NT structure (top), and basic electrostatic surface distribution (bottom), calculated using the vacuum electrostatics program in Pymol, version 0.99rc6. Red represents residues with negatively charged sidechain and blue indicate positively charged amino acids. **(B)** Topologies of Gp44NT (top) and Xis (bottom). **(C)** Superimposed Gp44_NT (green) and Xis/DNA complex (cyan, PDB ID: 2OG0, DNA major and minor grooves included). **(D)** Left: Overlay of 2D ^1^H–^15^N HSQC spectra of Gp44NT-His with and without dsDNA. Black: no dsDNA; Blue: 0.5 equivalent of dsDNA added and Red: 1 equivalent of dsDNA added. Spectra recorded at pH 6.5, 298K. Right: Overlay of 2D ^1^H–^15^N HSQC spectra of His-Gp44^1– 55^ with and without dsDNA. Black: no dsDNA and Red: 1 equivalent of dsDNA added. Spectra recorded at pH 6.5, 298K. **(E)** A model of Gp44NT/dsDNA interaction created by using Xis/DNA complex structure (PDB ID: 2IEF). Peaks experience most chemical shift perturbation is highlighted in red.

We determined its solution structure of Gp44^1–55^ using standard multidimensional NMR spectroscopy (PDB ID: 6L6V and BMRB ID: 36290). A notable feature in the solution structure of Gp44^1–55^ is that surface-exposed positively and negatively charged residues are located on opposite sides of the protein (Figure 3A). This type of charge distribution, especially with the positively charged residues on an α helix (α2 in Gp44^1–55^) resembles the charge distribution seen in a helix-turn-helix DNA binding motif. Indeed, a search for protein structure similarities using PDBefold (Krissinel and Henrick, 2005) revealed that the overall structure of Gp44^1–55^ exhibits a statistically significant similarity to an unusual DNA binding motif found in bacteriophage Lamda Xis protein—a DNA binding excisionase (Abbani et al., 2007). Xis adopts an unusual winged-helix structure in which two α helices are packed against two extended strands and similar positioning of α1 and α2 also found in Gp44^1–55^ (Figure 3B). In Xis, the four-residue linker between the two-stranded anti-parallel beta-sheet is called the “wing” (Figure 3B). Although the wing is “missing” in Gp44^1–55^, the N terminal region of Gp44^1–55^ bends back to α2 forming a loop to potentially mimic the Xis wing. When Xis interacts with DNA, α2 inserts into the major groove, while the wing contacts the adjacent minor groove and phosphodiester backbone. The overlay of Xis and Gp44^1–55^ with DNA suggests that Gp44^1–55^ could interact with DNA in a similar manner to Xis (Figure 3C).

To determine whether Gp44^1–55^ can indeed interact with DNA, we performed NMR titration experiments with two different double-stranded DNA probes containing strong promoters recognized by the *E. coli* or *B. subtilis* RNAP (Supplementary Table 2). Results revealed that Gp44^1–55^ binds double stranded DNA without displaying any preference for either DNA probe (Figure 3D). Overall, we conclude that the amino terminal region of Gp44 constitutes a DNA binding motif and build a Gp44NT/DNA complex model based on the published Xis/DNA complex structure and our NMR data (Figure 3E). Another interesting observation is that N-terminal (NT) His-tag block Gp44^1–55^ interaction with dsDNA (Figure 3D), perhaps by occluding the positive charged patch on Gp44 and inhibiting the interaction. Consistent with this, NT His-tagged Gp44 does not inhibit bacterial growth, indicating DNA binding is crucial for the function of Gp44 (Supplementary Figure 2).

### Domain 56–122 Mimics Single Strand DNA

**FIGURE 4:**
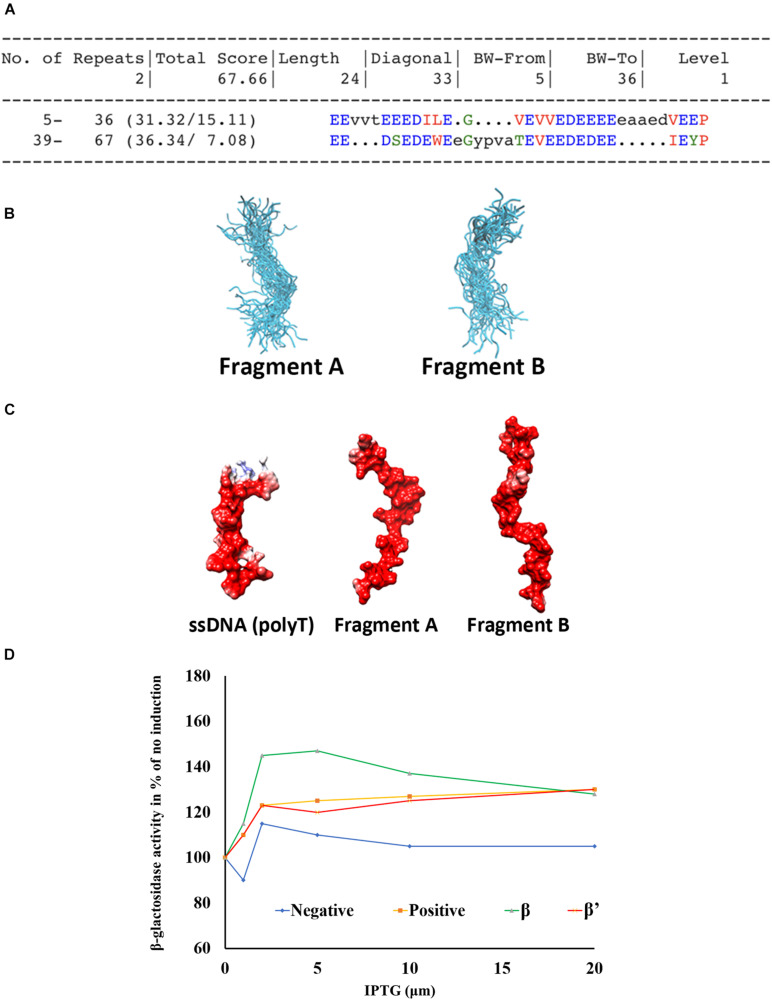
Domain 56–122 of Gp44 mimics single strand DNA. **(A)** Two conserved repeats (Fragment A and B) of Gp44^56– 122^ were extracted using RADAR. **(B)** The cartoon representation of the ensembles of disordered conformations populated by fragments A and B during the simulation. **(C)** The negatively charged and highly flexible nature of fragment A and B is comparable to that of single-stranded DNA—poly-Thymidine (Left). **(D)** BTH assay shows Gp44^56– 122^ interacts with β and β′ subunits *of B. subtilis*.

As the central region of Gp44 is predicted as disordered with significant negative charge and the equivalent region in σ^54^ (44–113) is mostly undefined random coiled yet contacting both β and β′ in the crystal structure of σ^54^-RNAP holoenzyme (Tabib-Salazar et al., 2019), we chose molecular dynamics to simulate this region to see is any interesting conformations are sampled and stable within the ensemble. First, we were able to extract two similarly conserved fragments from this region using RADAR (Li et al., 2015; Figure 4A) for use in simulations. These two fragments were simulated for 500 ns each under the updated CHARMM36 force field, optimized for both ordered and disordered proteins (Huang et al., 2017). The two fragments retain their random coil configuration throughout the simulations, not forming any secondary structure elements at any stage. The repulsion between sidechains of the highly charged segments prevents the formation of tertiary contacts. A representation of the ensembles of disordered conformations populated by fragments A and B during the simulations is shown in Figure 4B. The negatively charged and highly flexible nature of these fragments is comparable to that of single-stranded DNA (poly-thymidine) (Figure 4C), potentially suggesting that, in addition to DNA binding, DNA mimicry, is involved in the mode of action of Gp44. Flexibility is one of the interesting features for Gp44 as all previous reported DMPs do not have DNA-like flexibility due to rigid tertiary structure of proteins. Our attempts to express recombinant Gp44^55–122^ to conduct *in vitro* assays also failed, but BTH assays showed that this region alone is able to interact with β and β’ subunit of *B. subtilis* (Figure 4D). Interestingly, Gp44^1–122^ still has inhibitory effects although not as potent as the full length Gp44 in the growth attenuation assays (Supplementary Figure 2). These observations suggest that the construct with just a DNA binding motif which docks Gp44 on host genome and a DNA mimic region which binds to RNAP is capable of inhibiting host growth.

### Gp44CT Is Required for Optimal RNAP Inhibition

Our 1D NMR analysis shows that the C-terminal region does not possess any folded domains. Compared to the full length Gp44 NMR spectrum, line widths of folded domain peaks at 0.5 ppm increases and the relative intensity of these peaks compared with unstructured peaks decreases for Gp44^1–122^ despite being a shorter construct, suggesting the C-terminus may alleviate aggregation. This region possesses both positive and negative charges in mosaicking blocks, which may play a role in preventing self-tangling (Weiss and Shaw, 2015) keeping the DNA mimic domain accessible (Figure 2C). The inhibitory effect of Gp44ΔCT (Gp44^1–122^) is also affected (Supplementary Figure 2), suggesting it is also required for optimal inhibition of the host transcription by this phage protein. Consistent with this, we observed less response of domain 56–122 compared to full length Gp44 in the BTH assay (Figure 4D), also indicating that C-terminal is required for its optimal function.

## Discussion

Gp44 has some interesting features. First, its NT DNA binding motif resembles the fold adopted by Lamda phage protein Xis, which is part of the excision complex essential for the excision of prophage from the host genome *via* site-specific recombination. Gp44^1–55^ has a further simplified structure at DNA minor grove interactive part and perhaps as a consequence, it gives up any sequence specificity. Importantly, DNA binding is essential for the inhibitory effect on host cell growth, possibly because the DNA binding property increases the chance to encounter RNAP. The middle section Gp44^56–122^ also has some unique features. It is extremely negatively charged, and it is likely that this region would be susceptible to be interacting with various nucleic acids binding protein in the bacterium. The DNA mimicry is provided by a flexible disordered domain that possesses the ability to adopt and resemble a single strand DNA-like conformation. Although the unstructured C-terminal domain remains largely uncharacterized, it is also essential for inhibition of the host transcription, possibly by preventing self-entanglement.

**FIGURE 5:**
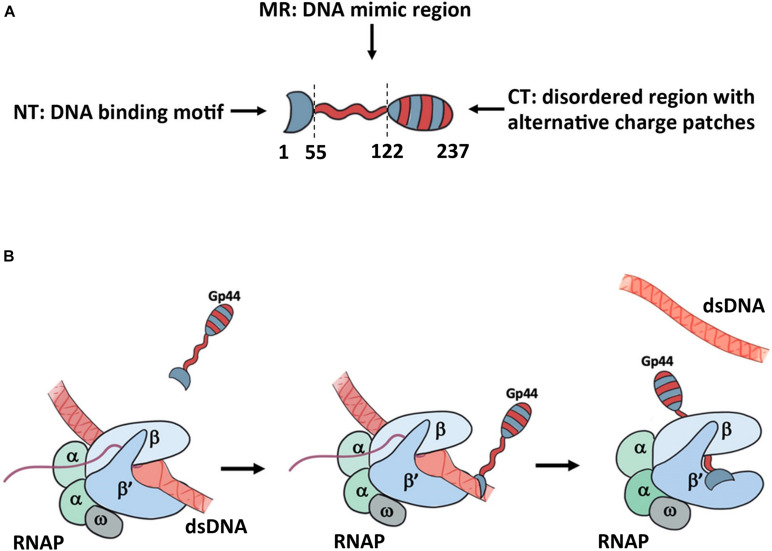
Proposed model for the mechanism of action for Gp44. **(A)** the domain arrangement of Gp44 in cartoon. Red stands for negatively charged and blue stands for positively charged patch. **(B)** Proposed model for Gp44/DNA/RNAP interaction. While the exact mechanism of how Gp44 replaces DNA remains unclear, it could explain previous observations, including causing premature of mRNAs.

With a NT DNA binding motif, a DNA mimic domain and the CT putative self-protection domain, Gp44 has an unusual domain organization (Figure 5A). Our growth attenuation assays show that none of these domains is able to inhibit the host growth alone and removal of any domain from Gp44 abolishes or decrease its ability to kill the bacteria (Figure 5A). Thus, we proposed a model for mechanism of action on inhibition of host RNA synthesis by Gp44 inspired by σ^54^ (Figure 5B). In our model, Gp44 first docked to host dsDNA, waiting for the arrival of transcribing host RNAP. When RNAP contacts the Gp44 during the transcriptional elongation, Gp44 slips into the transcription bubble with its DNA mimic region interacting with β and β subunit. Once Gp44 occupied RNAP, its CT stops Gp44 slipping away and results in a lockdown on RNAP, causing mRNA premature as described in previous studies. Our proposed model is supported by the broad range bacterial inhibition capability. Potentially, Gp44 might work as the universal inhibitory for many if not all bacteria. With further optimization, it could be even incorporated into existing bacteriophages to enhance their anti-bacteria activity thus used in phage therapy.

## Data Availability Statement

The datasets presented in this study can be found in online repositories. The names of the repository/repositories and accession number(s) can be found below: http://www.wwpdb.org/, 6L6V.

## Author Contributions

SW and BL designed the research. BL, NM, ZW, HW, MS-H, JM, and YW performed the research. SW, SM, and BL wrote the manuscript. All authors contributed to the article and approved the submitted version.

## Conflict of Interest

The authors declare that the research was conducted in the absence of any commercial or financial relationships that could be construed as a potential conflict of interest.
