# Supplementary material for: A Bacteriophage DNA Mimic Protein Employs a Non-specific Strategy to Inhibit the Bacterial RNA Polymerase
Source: Front Microbiol. 2021 Jun 2;12:692512. doi: 10.3389/fmicb.2021.692512 (PMC8208478; doi:10.3389/fmicb.2021.692512)
Supplement: Supplementary file 1 [file Table_1.DOCX]

**Figure S1: Biochemical characterization of Gp44 and its sequence alignment with σ^54^.**

**(A)** **ITC results for the affinity determinations of Gp44-β and Gp44-** **β', respectively. (B) Gp44(56-122) sequence aligned with σ^54^ (44-113) by NEEDLE.**

**A**
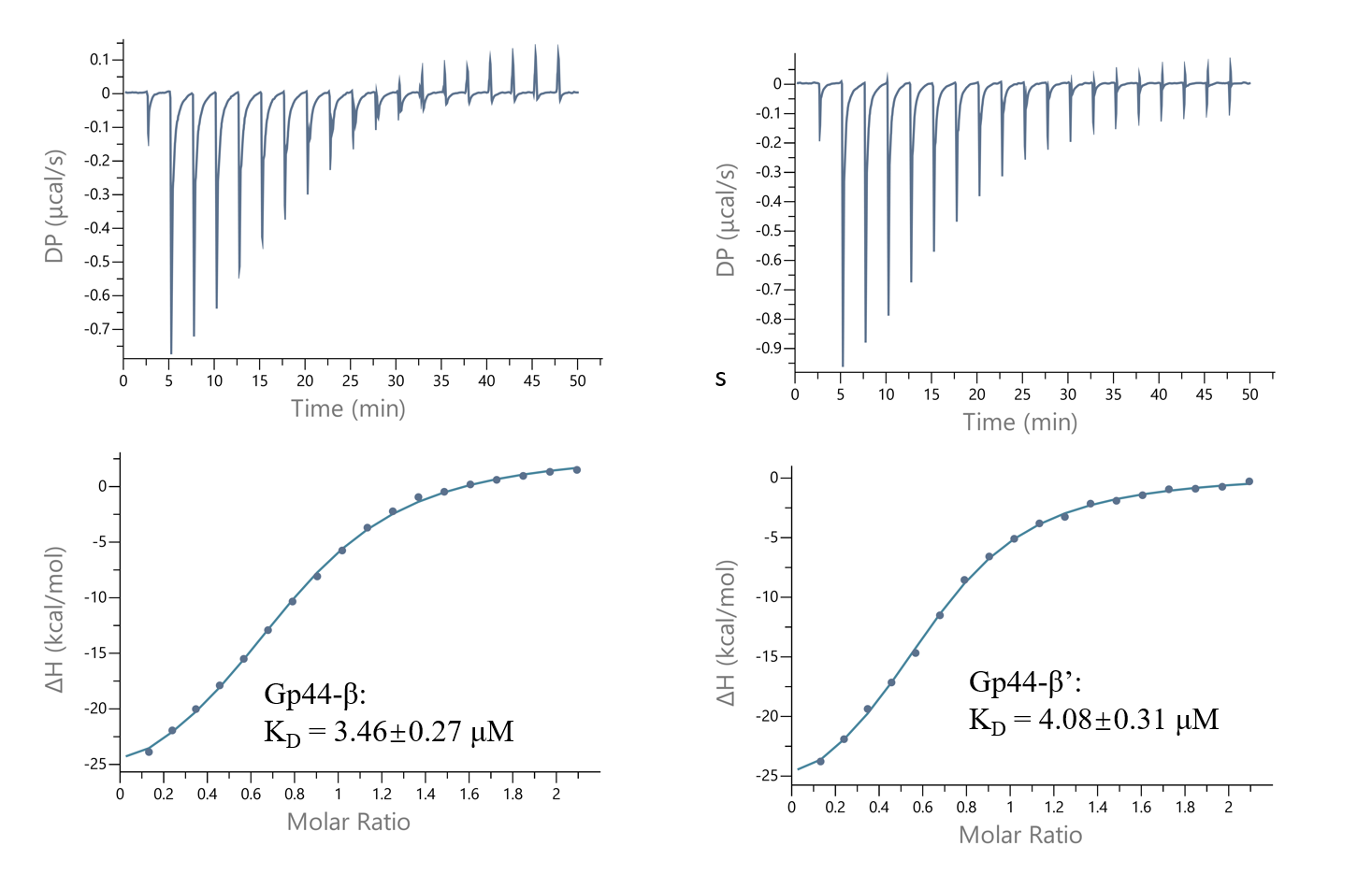


**B**

**Gp44(56-122) 1 EEVVEEVVTEEE-DILEGVEVVEDEEEEEAAEDVEEPTSEED--SEDEWE 47**

**..::|::.|.|| |..| .:|.|..:.|:.:|:....|: .:..|:**

**σ54(44-113) 1 NPLLEQIDTHEEIDTRE----TQDSETLDTADALEQKEMPEELPLDASWD 46**

**Gp44(56-122) 48 ----EGYPVATEVEEDEDEEIEYP 67**

**.|.|..|..:..:||...|.**

**σ54(44-113) 47 TIYTAGTPSGTSGDYIDDELPVYQ 70**

**Figure S2: Growth attenuation assay: Graphs showing growth curves of *E. coli* cultures expressing different constructs of Gp44 and that of control cultures of empty pDE2 vector (see figure legend).**


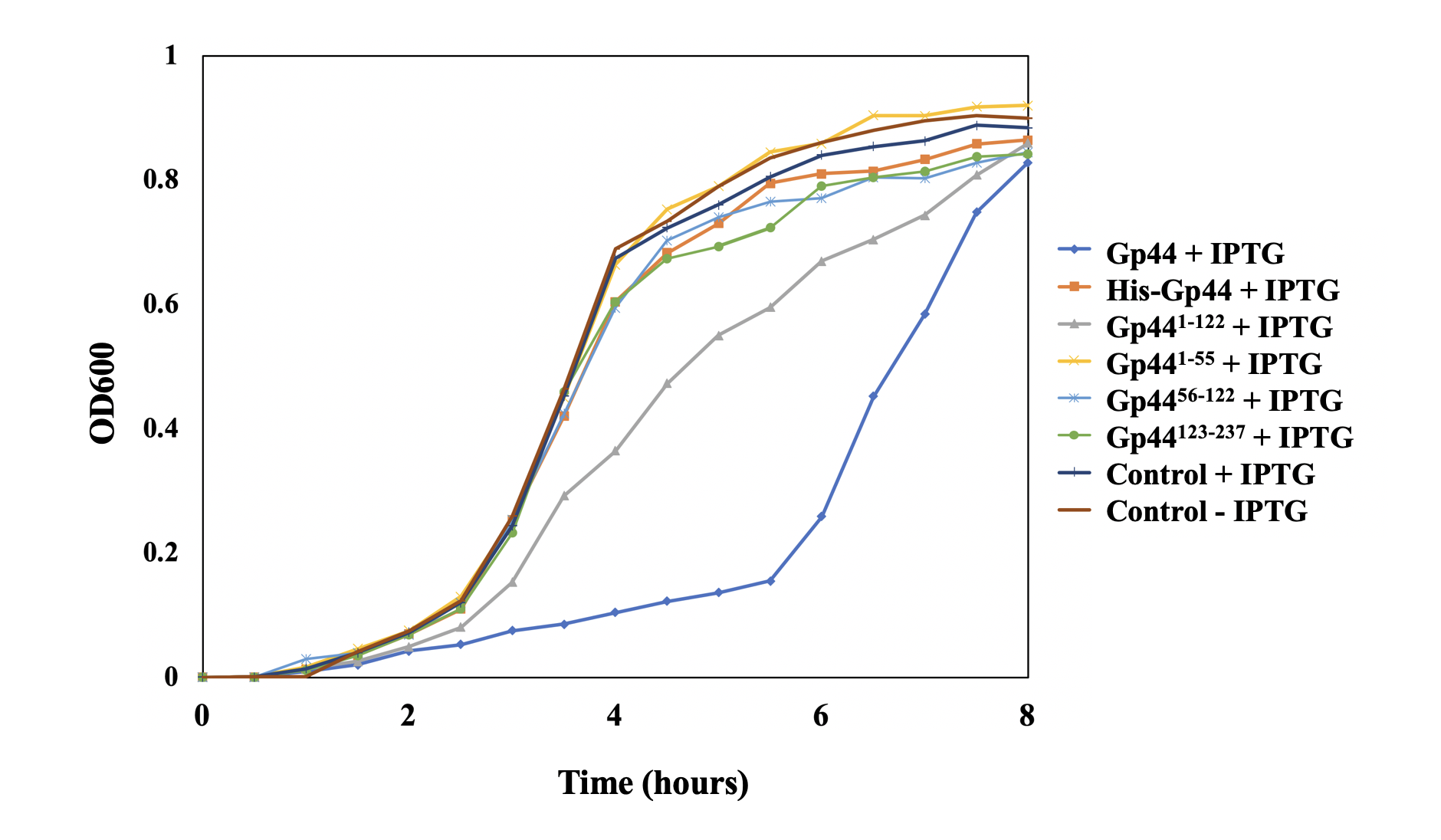


**Table S1: DNA fragments used in NMR titration experiment.**

| Name | Description | Sequence | Length |
| --- | --- | --- | --- |
| Pstrong  (BBa_K780003) | Strong constitutive promoter for Bacillus subtilis | aattgcagtaggcatgacaaaatggactca | 30 |
| T7A1  (BBa_I712074) | T7A1 promoter used by T7 polymerase in Escherichia coli | agggaatacaagctacttgttctttttgca | 30 |

**Table S2: Structural statistics from the solution structure calculation for 6xHis Gp44 N-terminal region (1-55) (PDB ID: 6L6V, BMRB ID: 36290).**

| Gp5.7 | Value |
| --- | --- |
| NMR Distance and Dihedral Constraints |  |
| Distance constraints |  |
| Total NOE | 875 |
| Intraresidue | 354 |
| Interresidue | 521 |
| Sequential (\|*i-j*\|)=1) | 181 |
| Short range (2≤\|*i-j*\|)≤3) | 87 |
| Medium range (4≤\|*i-j*\|)≤5) | 56 |
| Long range (\|*i-j*\|)>5) | 197 |
| Total Dihedral angle Restraints | 84 |
| φ | 42 |
| ψ | 42 |
| Total RDCs | 0 |
| Structural Statistics |  |
| Violations (mean and SD) |  |
| Distance constraints (Å) | 0.024 ± 0.007 |
| Dihedral angle constraints (°) | 0.30 ± 0.073 |
| Maximum dihedral angle violation (°) | 0.64 |
| Maximum distance constraint violation (Å) | 0.22 |
| Deviations from idealized geometry |  |
| Bond length (Å) | 0.0015 ± 0.000 |
| Bond angle (°) | 0.319 ± 0.006 |
| Impropers (°) | 0.263 ± 0.011 |
| Average Pairwise rmsd^α^(Å) |  |
| Heavy | 0.576 ± 0.0843 |
| Backbone | 0.216 ± 0.0470 |

**Table S3: Parameters for molecular dynamics simulation of each fragments from Gp44^56-122^**

| **MD simulation parameters** | | |
| --- | --- | --- |
| Integrator | md | Leap-frog integrator |
| Nsteps | 250000000 | 2 * 250000000 = 500 ns |
| dt | 0.002 | fs |
| **Output control** | | |
| nstxout | 0 |  |
| nstvout | 0 |  |
| nstfout | 0 |  |
| nstenergy | 250000 |  |
| nstlog | 250000 |  |
| nstxout-compressed | 250000 |  |
| compressed-x-grps | system |  |
| **Bond parameter** | | |
| continuation | yes | Restarting after NPT |
| constrain algorithm | LINCS | holonomic oncstrains |
| constrains | H-bonds | Bonds involving H are constrained |
| LINCS iteration | 1 |  |
| LINCS order | 4 |  |
| **Neighbor searching** | | |
| cutoff-scheme | Verlet |  |
| ns type | grid |  |
| nstlist | 10 |  |
| rcoulomb | 1.0 nm |  |
| rvdw | 1.0 nm |  |
| **Electrostatics** | | |
| coulomb type | Particle Mesh Ewald for long-range electrostatics (PME) | |
| PME order | 4 | cubic interpolation |
| fourier spacing | 0.16 |  |
| **Temperature Coupling** | | |
| temperature coupling | V-rescale | modified Berendsen thermostat |
| temperature coupling group | Protein | Non protein |
| τ(t) time constant | 0.1 | 0.1 |
| reference temperature (K) | 300 | 300 |
| **Pressure Coupling** | | |
| pressure coupling | Parrinello-Rahman |  |
| pressure coupling type | isotropic |  |
| τ(t) time constant | 2.0 |  |
| reference pressure (bar) | 1.0 |  |
| compressibility (bar^-1^) | 4.5e-05 | isothermal compressibility of water |
